# Phylogenetic analyses reveal that *Schellackia* parasites (Apicomplexa) detected in American lizards are closely related to the genus *Lankesterella*: is the range of *Schellackia* restricted to the Old World?

**DOI:** 10.1186/s13071-017-2405-0

**Published:** 2017-10-10

**Authors:** Rodrigo Megía-Palma, Javier Martínez, Dhanashree Paranjpe, Verónica D’Amico, Rocío Aguilar, María Gabriela Palacios, Robert Cooper, Francisco Ferri-Yáñez, Barry Sinervo, Santiago Merino

**Affiliations:** 1 Departamento de Ecología Evolutiva, Museo Nacional de Ciencias Naturales-CSIC, Madrid, Spain; 2 Departamento de Biomedicina y Biotecnología, Área de Parasitología, Universidad de Alcalá de Henares, Alcalá de Henares, Spain; 3 Department of Ecology and Evolutionary Biology, University of California, Santa Cruz, California 95064 USA; 4 Department of Biodiversity, Abasaheb Garware College, Pune, India; 5 Grupo de Ecofisiología Aplicada al Manejo y Conservación de la Fauna Silvestre, Centro para el Estudio de Sistemas Marinos, Centro Nacional Patagónico, Puerto Madryn, Chubut Argentina; 6 Instituto Argentino de Zonas Áridas, Grupo de Investigaciones de la Biodiversidad CONICET MENDOZA, Mendoza, Argentina; 7 School of Biosciences, The University of Melbourne, Melbourne, VIC Australia; 8 Departamento de Biogeografía y Cambio Global, Museo Nacional de Ciencias Naturales-CSIC, Madrid, Spain

**Keywords:** Haemococcidia, Lankesterellidae, *Lankesterella*, Schellackiidae, *Schellackia*, Reptile

## Abstract

**Background:**

Species of *Schellackia* Reichenow, 1919 have been described from the blood of reptiles distributed worldwide. Recently, *Schellackia* spp. detected in European and Asian lizards have been molecularly characterised. However, parasites detected in American lizard hosts remain uncharacterised. Thus, phylogenetic affinities between the Old and New World parasite species are unknown.

**Methods:**

In the present study, we characterised morphologically and molecularly the hemococcidian parasites (sporozoites) that infect three lizard hosts from North America and two from South America.

**Results:**

In total, we generated 12 new 18S rRNA gene sequences of hemococcidian parasites infecting New World lizard hosts. By the microscopic examination of the smears we identified *Schellackia golvani* Rogier & Landau, 1975 (ex *Anolis carolinensis* Voigt) and *Schellackia occidentalis* Bonorris & Ball, 1955 (ex *Uta stansburiana* Baird & Girard and *Sceloporus occidentalis* Baird & Girard) in some samples, but the phylogenetic analysis indicated that all 18S rDNA sequences are distant from *Schellackia* species found in Old World lizards. In fact, the hemococcidian parasites detected in the New World lizards (including *S. occidentalis* and *S. golvani*) were closely related to the genus *Lankesterella* Labbé, 1899. Consequently, we suggest these two species to be included within the genus *Lankesterella*.

**Conclusions:**

Life history traits of hemococcidian parasites such as the type of host blood cells infected, host species or number of refractile bodies are not valid diagnostic characteristics to differentiate the parasites between the genera *Schellackia* and *Lankesterella*. Indeed, lankesterellid parasites with a different number of refractile bodies had a close phylogenetic origin. Based on the phylogenetic results we provide a systematic revision of the North American hemococcidians. Our recommendation is to include the species formerly described in the genus *Schellackia* that infect American lizards into *Lankesterella* (Lankesterellidae) as *Lankesterella golvani* (Rogier & Landau, 1975) n. comb and *L. occidentalis* (Bonorris & Ball, 1955) n. comb.

**Electronic supplementary material:**

The online version of this article (10.1186/s13071-017-2405-0) contains supplementary material, which is available to authorized users.

## Background

*Lankesterella* Labbé, 1899 [1] and *Schellackia* Reichenow, 1919 [2] are two genera of haemococcidian parasites of independent evolutionary origin that are nested within a paraphyletic Eimeriidae [3]. The evolutionary novelty in the life-cycle of parasites in these genera is the participation of a blood-sucking vector that exerts a mechanical role in the transmission between hosts. Therefore, the infective stages of the protozoans remain dormant in the transmitter without undergoing any development or modification [4]. Thus, at least in lizard hosts, transmission is accomplished by predation on the infected invertebrate (but see [5]). Some authors consider *Lainsonia* Landau, 1973 [6] as a third genus of hemococcidia that undergoes sporogony within reticuloendothelial host cells of the liver, spleen, lungs, kidney, and capillaries of the brain [4] in South American lizard host species [4, 7]. However, other authors prefer to consider *Lainsonia* as a synonym of *Schellackia* based on the common characteristic of their life-cycles, i.e. the presence of eight sporozoites in the oocyst [4, 8].

The genus *Schellackia* was originally described in European lizard hosts [2]. So far, species of *Schellackia* have been described infecting frog and lizard hosts from Europe, America, Asia and Africa [9–14]. *Schellackia* sp. was reported to infect the scincid lizard *Egernia stokesii* in Australia [15] but the identification of these parasites remains to be molecularly confirmed. The second hemococcidian genus, *Lankesterella*, was erected for a species infecting frogs [1]. Later, species of this genus were described infecting birds and lizards [3, 16–23]. The main morphological difference used to classify the hemococcidia into *Lankesterella* or *Schellackia* has been the characteristics of the oocyst during the endogenous development of the parasite. The presence of oocysts, normally in the *lamina propia* of the intestine, with eight naked sporozoites surrounded by a soft-walled oocyst, has been considered a diagnostic character for the genus *Schellackia*. In contrast, the presence of 32 or more sporozoites is the diagnostic characteristic for the genus *Lankesterella* [4]. However, the latter was true for *Lankesterella* spp. described in anuran hosts [8, 18, 21], since no endogenous development of *Lankesterella* spp. infecting lizard hosts has been described so far. The apicomplexan genera *Lankesterella* and *Schellackia* were largely believed to form a monophyletic clade within the family Lankesterellidae [4, 8]. However, phylogenetic analyses revealed they have an independent evolutionary origin [3].

In lizards, there are 12 species of the genus *Schellackia* described worldwide [4]. Five of these were described from lizards in the Americas. Among these American species, three occur in Brazil (i.e. *Lainsonia* spp.) and two occur in North America (*S. golvani* Rogier & Landau, 1975 and *S. occidentalis* Bonorris & Ball, 1955). The other seven parasite species, *S. orientalis* Telford, 1993 [24], *S. calotesi* Finkelman & Paperna, 1998 [25], *S. bolivari* Reichenow, 1919 [2], *S. brygooi* Landau, 1973 [6], *S. ptyodactyli* Paperna & Finkelman, 1996 [13], *S. agamae* Bristovetzky & Paperna, 1990 [26] and *S. bocagei* Álvarez-Calvo, 1975 [27] were detected in Old World lizards [4]. The sporozoites show one refractile body (RB) in *S. bolivari*, *S. bocagei, S. orientalis*, *S. brygooi, S. golvani* and *S. legeri* (syn. *Lainsonia legeri*) [3, 4], whereas *S. calotesi*, *S. ptyodactyli*, *S. agamae, S. occidentalis*, *S. landaue* and *S. iguanae* (syn. *Lainsonia iguanae*) show a variable number of RB (between zero and two [4]). All these species were described infecting mainly erythrocytes, but also can infect lymphocytes and monocytes [4]. The exception is *S. golvani* that characteristically infects polymorphonuclear leukocytes [4, 10]. On the other hand, information on the *Lankesterella* parasites that infect reptiles is scarce. Only two species of *Lankesterella*, *L. millani* Álvarez-Calvo, 1975 [27] and *L. baznosanui* Chiriac & Steopoe, 1977 [28], were described infecting lizard hosts in the world. However, according to Telford [4] these two taxa were not further considered as valid species because they were originally described based on insufficient morphological data.

No information on the molecular diversity and the phylogenetic affinities of the hemococcidia that infect New World lizard hosts is available. Therefore, we have sampled different populations of New World lizards belonging to the families Phrynosomatidae (genera *Uta* Baird & Girard and *Sceloporus* Wiegmann), Iguanidae (genus *Dipsosaurus* Hallowell), and Liolaemidae (genera *Liolaemus* Wiegmann and *Phymaturus* Gravenhorst) to obtain molecular data that allowed the study of the evolutionary affinities between hemococcidia detected in Old- and New World host lizards. In addition, we present morphological data on the sporozoites found infecting the blood of the American lizard hosts studied here.

## Methods

### Sampling methods

We gathered several blood samples with hemococcidian parasites that were collected in different localities in North and South America. From California, blood was obtained from six infected *Uta stansburiana hesperis* Richardson (Phrynosomatidae) from Corn Springs (South California) and 12 from Los Baños (West California), 19 *Sceloporus occidentalis bocourtii* Boulenger (Phrynosomatidae) from Santa Cruz (West California), two *S. occidentalis biseriatus* Hallowell from high elevation (1800 masl) from the Sierra Nevada mountains (CA, USA) and one *Dipsosaurus dorsalis* Baird & Girard (Iguanidae) from Boyd Canyon, Riverside County (South California). One infected sample of *Liolaemus pictus* Müller & Hellmich (Liolaemidae) from Huinay (Chile) was also included in the study. All authors captured the reptiles using a noose tied to the end of a fishing pole (e.g. [3, 14]). All animals were processed and released on the same spot where they had been captured.

Blood samples were collected at the base of the tail using individual sterile needles (BD Microlance, Huesca, Spain). In the case of male lizards, the hemipenes bulges were always avoided to prevent harming the individuals. The area where the blood sample was obtained was previously cleaned with ethanol 96%. Blood droplets were collected with the help of Na-heparinized microhematocrit tubes (BRAND, Wertheim, Germany). Blood samples were preserved in two different ways. First, thin blood smears were made for microscopic examination of infection. Blood smears were air-dried and fixed for 5 min in absolute methanol. All blood smears were stained with Giemsa stain (1/10 *v*/v) for 40 min. Slides were examined by the same operator for hemoparasites counting to 15,000 red blood cells (RBC) following Merino & Potti [29]. We made microphotographs of the sporozoites observed under light microscopy and measured the length and the width of the sporozoites using the MB-ruler 5.0 free software (http://www.markus-bader.de/MB-Ruler/index.php) [3, 14]. Thereafter, we performed an ANOVA analysis to compare the size of the sporozoites that we detected in the two known hosts for *Schellackia occidentalis*: *Sceloporus occidentalis* and *U. stansburiana*. Secondly, the remaining blood (less than 20 μl) was preserved on Whatman FTA Classic Cards (GE Healthcare UK Limited, Buckinghamshire, UK). These cards allowed us to perform DNA extraction later [3, 14, 23].

D’Amico & Aguilar [30] reported the presence of hemococcidian parasites in the blood of *Phymaturus payuniae* Cei & Castro (Liolaemidae) from the cold desert in Patagonia, Argentina. These authors provided a tissue sample (tail tip) of one infected individual preserved in 70% ethanol for this study. Tail tissue samples allow the proper extraction of the apicomplexan DNA from the host tissue [31]. Similarly, we microscopically analysed the blood smear corresponding to the infected polychrotid lizard, *Anolis carolinensis*, reported in a previous study [23]. The infected lizard was one individual recently imported from Florida [23] that presented an intensity of infection of 21/15,000 RBC. In the present study, we compared the morphology of parasites found in the American lizards of genera *Anolis* [23], *Sceloporus*, *Uta*, *Dipsosaurus*, *Phymaturus* [30] and *Liolaemus* with previous species described infecting American lizard hosts using the morphological data available in Telford [4].

### Molecular methods

We extracted genomic DNA from the blood preserved on FTA cards following the protocol described by Megía-Palma et al. [14]. The DNA was then purified using the NZYGelpure kit (NZYTech, Lda., Lisbon, Portugal). Partial amplification of the 18S ribosomal RNA gene sequence was performed using the primer set BT-F1/hep1600R or BT-F1/EimIsoR1 or BT-F1/EimIsoR3 (Table 1). These primers were previously used to amplify other coccidian species [3, 23]. PCR reactions (total volume of 20 μl) contained between 20 and 100 ng of the DNA template. Supreme NZY*Taq* 2× Green Master Mix (NZYTech, Lda.) and 250 nM of each primer were used. Using a Veriti thermal cycler (Applied Biosystems, Foster City, CA, USA), reactions were run using the following conditions: 95 °C for 10 min (polymerase activation), 40 cycles at 95 °C for 30 s, annealing temperature at 58 °C for 30 s, 72 °C for 120 s, and a final extension at 72 °C for 10 min. All amplicons were recovered from agarose gels and subjected to direct sequencing using an ABI 3730 XL automated sequencer (Applied Biosystems). Amplicons whose chromatograms yielded double peaks were cloned using NZY-A Speedy PCR cloning kit (NZYTech, Lda.). Plasmids were purified using NZYMiniprep kit (NZYTech, Lda.).

**Table 1:** Primer sets used to detect haemococcidians in the present study

Primer	Sequence 5′– 3′	Amplicon size (bp)
BT-F1	GGTTGATCCTGCCAGTAGT	1626
Hep1600R	AAAGGGCAGGGACGTAATCGG	
BT-F1	As above	1580
EimIsoR1	AGGCATTCCTCGTTGAAGATT	
BT-F1	As above	1528
EimIsoR3	GCATACTCACAAGATTACCTAG	

Due to the weak signal obtained by PCR in some samples, we could only sequence 22 of the 41 amplicons that we obtained. The twelve different DNA sequences (18S rRNA gene) obtained in the present study were aligned together with other 98 sequences obtained from GenBank. The alignment was performed using PROBCONS (http://toolkit.tuebingen.mpg.de/probcons). Poorly aligned positions and divergent regions of the alignment were suppressed using the GBlocks program [32] selecting the following options: minimum number of sequences for a conserved position = 56; minimum number of sequences for a flank position = 56; maximum number of contiguous nonconserved positions = 10; minimum length of a block = 5; and allowed gap positions = with half. The final alignment contained 1441 positions and 110 sequences. The substitution model GTR + I + G was selected using jModeltest 2.1.4 [33] to perform the Bayesian analysis with MrBayes 3.2.6 software [34–36]. This analysis consisted of 2 runs of 4 chains each with 2,000,000 generations per run and a sampling interval every 100 generations. After a ‘burn-in’ of 500,000 generations was applied and 30,000 trees were obtained for consensus tree. The final standard deviation of the split frequencies was lower than 0.01. In addition, the alignment was also analysed using a maximum likelihood inference (PhyML 3.0 software) [37], using the substitution model listed above. The subtree pruning and regrafting (SPR) tree rearrangement option were selected, and a Bayesian-like transformation of aLRT (aBayes) was used to obtain the clade support [38]. Both trees were rooted with the family Sarcocystidae because this taxon is well established as a distinct but closely related family to the Eimeriidae [8, 14].

### ZooBank registration

To comply with the regulations set out in article 8.5 of the amended 2012 version of the *International Code of Zoological Nomenclature* (ICZN) [39], details of the article have been submitted to ZooBank. The Life Science Identifier (LSID) of the article is urn:lsid:zoobank.org:pub:44981DFE-E5A2-446C-8865-DC90B73C90CD.

## Results

### Morphological identification

**Fig. 1:**
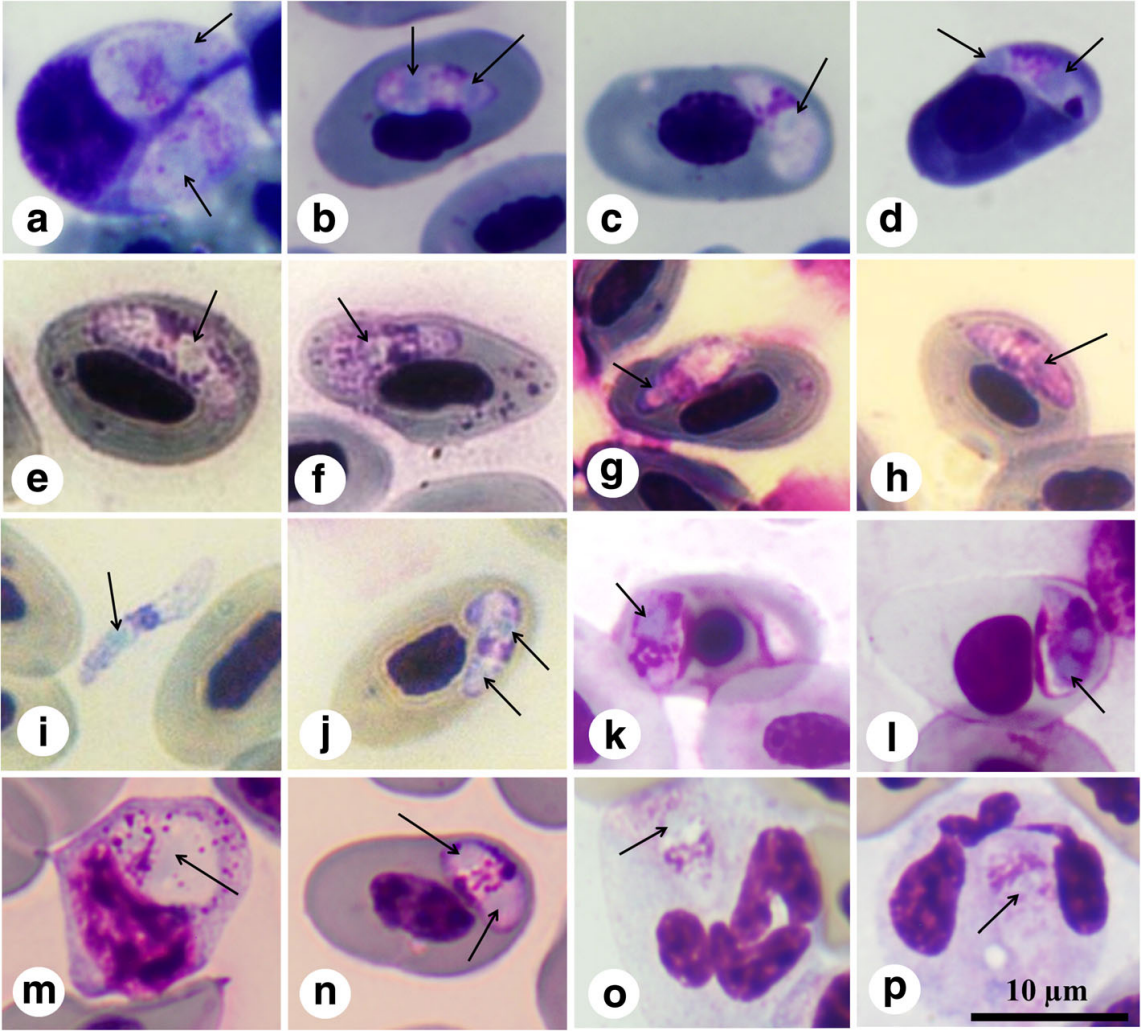
Microphotographs of hemococcidian parasites infecting the blood of New World lizard hosts. Every pair of microphotographs corresponds to one specific haplotype as follows: **a**, **b** and **c**, **d**: SO1 and SO2 in *Sceloporus occidentalis*, respectively; **e**, **f**; **g**, **h**; and **i**, **j**: US1, US3 and US4 in *U. stansburiana*, respectively; **i** free sporozoite in the blood of *U. stansburiana*; **k**, **l:** LP1 in *L. pictus*; **m**, **n**: DD in one lymphocyte (**m**) and one erythrocyte (**n**) of *D. dorsalis*; **o**, **p** *Lankesterella* sp. Lank_anocar in polymorphonuclear leukocytes of *A. carolinensis*. Arrows indicate RB in the sporozoites. All pictures were made at the same scale. *Scale-bar*: 10 μm

In total, 99% (207/209) of the observed sporozoites had light blue refractile bodies (RB) (Fig. 1). The presence of RB is a morphological characteristic compatible with the morphology of the sporozoites in the hemococcidian genera *Lankesterella* and *Schellackia* [3, 4]. The number of RB in each of the parasite haplotypes is shown in Table 2. We observed 5% (11/209) of the sporozoites free in the plasma, whereas the remaining 95% (198/209) of the sporozoites were found infecting the cytoplasm of host blood cells. More, specifically 65% of the sporozoites infected red blood cells, and 30% leukocytes. Host blood cells for the sporozoites of each parasite haplotypes are indicated in Table 2.

**Table 2:** Prevalence of each of the parasites haplotypes in the infected individuals of each host species, host cell type and number of RB for each of the described species of hemococcidia and parasite haplotypes that infect American lizards

Parasite haplotype or described species	Prevalence (%) (infected/examined)	Host species	Infected cell	RB
SO1	79 (11/14)	*Sceloporus occidentalis*	E & L	1–2
SO2	21 (3/14)	*S. occidentalis*	E & L	0–1
US1	66 (4/6)	*Uta stansburiana*	E	1
US2a, US2b^a^	17 (1/6)	*U. stansburiana*	E	1
US3	17 (1/6)	*U. stansburiana*	E	1–2
*Schellackia occidentalis* ^b^	–	*S. occidentalis* and *U. stansburiana*	E (primarily)	1–2
*Lankesterella* sp. Lank_anocar	–	*Anolis carolinensis*	PL	1
*Schellackia golvani* ^b^	–	*A. carolinensis*	PL	1
DD1, DD2, DD3, DD4^a^	100 (1/1)	*Dipsosaurus dorsalis*	E	1–2
LP1	100 (1/1)	*Liolaemus pictus*	E	1
PP1^c^	100 (1/1)	*Phymaturus payuniae*	E	1–2
*Schellackia landaue* ^b^	–	*Polychrus* spp.	E & L (M, Ly)	1–2
*Lainsonia iguanae* ^b^	–	*Iguana iguana*	E & L (M)	2
*Lainsonia legeri* ^b^	–	*Tupinambis nigropunctatus*	L (M, Ly)	1

*Abbreviations*: *E* erythrocyte, *L* leukocyte, *M* monocytes, *Ly* lymphocytes, *PL* polymorphonuclear leukocytes, *RB* number of refractile bodies

^a^Clone haplotypes US2a and US2b and clone haplotypes DD1, DD2, DD3 and DD4 were detected in a single *U. stansburiana* and a single *D. dorsalis*, respectively

^b^Morphological data from *Schellackia* species obtained from Telford [4]

^c^Data obtained from D’Amico & Aguilar [30]

The hemococcidian parasite found in the blood of *Anolis carolinensis* was morphologically identified as *Schellackia golvani*. The mean size of the sporozoites was 8.4 × 3.9 μm and all of them were observed in the cytoplasm of polymorphonuclear leukocytes (21/21) with a single RB.

Sporozoites of *Schellackia occidentalis* were detected in the cytoplasm of erythrocytes and leukocytes of the lizard host *Sceloporus occidentalis*, and only in the cytoplasm of erythrocytes of the lizard host *Uta stansburiana*. In addition, in both host species we found few sporozoites that were free in plasma. In *S. occidentalis*, 51.8% (57/110) of sporozoites were observed in the cytoplasm of leukocytes (mean size ± standard deviation, SD: 9.3 ± 1.6 μm × 4.2 ± 1.2 μm) (Fig. 1a); 42.7% (47/110) were observed in the cytoplasm of erythrocytes (mean size ± SD: 7.3 ± 1.2 μm × 3.5 ± 0.9 μm) (Fig. 1b), and 5.5% (6/110) were free in plasma (mean size ± SD: 8.6 ± 1.6 μm × 2.9 ± 0.5 μm). In addition, 2% (2/110) of the sporozoites presented 0 RB, 29% (32/110) presented a single one RB, and 69% (76/110) presented 2 RB. In *U. stansburiana* hosts, 81% (21/26) of the sporozoites infected erythrocytes (mean size ± SD: 9.2 ± 1.3 μm × 3.2 ± 0.7 μm) (Fig. 1e) and 9% (5/26) were free in plasma (mean size ± SD: 10.7 ± 1.0 μm × 2.4 ± 0.6 μm) (Fig. 1i). No sporozoite was observed infecting leukocytes. Besides, 73% (19/26) of the sporozoites presented 1 RB, and 27% (7/26) presented 2 RB. The sporozoites found in *U. stansburiana* were longer (*F*_(1,134)_ = 8.1, *P* = 0.005) and narrower (*F*_(1,134)_ = 12.2, *P* = 0.0006) than the sporozoites found in *S. occidentalis*. This result was independent of the type of cell infected (interaction: host species*type of cell infected: (length) *F*_(1,131)_ = 0.1, *P* = 0.73; (width) *F*_(1,131)_ = 0.02, *P* = 0.88).

### Molecular results

We performed DNA extraction from the 41 samples where we found hemococcidians infecting blood cells in blood smears. We only sequenced 22 of the 41 (53%) amplicons obtained. In total, we found 12 new haplotypes of hemococcidian parasites infecting New World lizard hosts (Table 3). The new sequence data are available in the GenBank database under the accession numbers MF167544–MF167555 (see also Additional file 1: Table S1). In North American hosts, we found six new haplotypes of the 18S rRNA gene of hemococcidian parasites infecting Phrynosomatidae and four infecting Iguanidae whereas, in South American hosts, we found two haplotypes infecting Liolaemidae lizards. The prevalence of each parasite haplotype in the host species sample is shown in Table 2.

**Table 3:** Lizard host species and localities where each parasite haplotype was found

Parasite haplotype	Clade	Host species	Locality
SO1, SO2	A	*Sceloporus occidentalis biseriatus* *Sceloporus occidentalis bocourtii*	Sierra Nevada and Santa Cruz, CA, USA
US1	A	*Uta stansburiana*	Corn Springs and Los Baños, CA, USA
US2a, US2b	A	*Uta stansburiana*	Corn Springs, CA, USA
US3	A	*Uta stansburiana*	Los Baños, CA, USA
DD1, DD4	C	*Dipsosaurus dorsalis*	Boyd Deep Canyon, CA, USA
DD2, DD3	A	*Dipsosaurus dorsalis*	Boyd Deep Canyon, CA, USA
LP1	A	*Liolaemus pictus*	Huinay, Chile
PP1	A	*Phymaturus payuniae*	Patagonia, Argentina

**Fig. 2:**
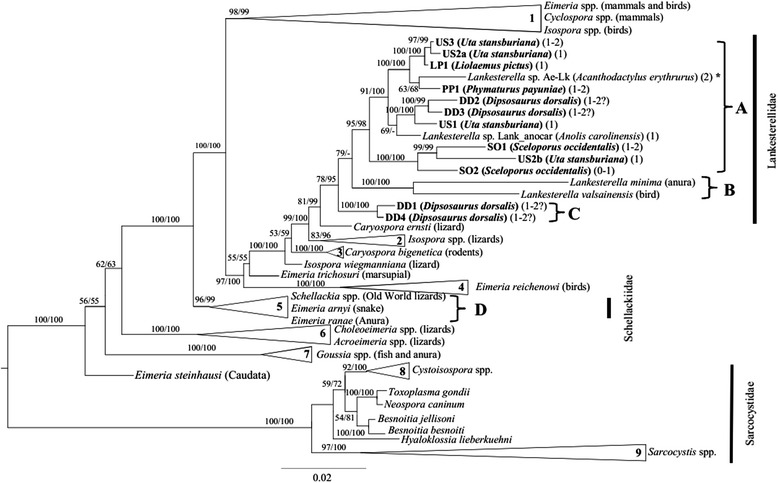
Phylogenetic tree derived from Bayesian inference (BI) and Maximum Likelihood (ML) analyses. Node support provided as BI/ML; a hyphen indicates no phylogenetic support. Hosts from which the sequences were obtained are listed in parentheses. The number of refractile bodies in sporozoites is shown in parentheses after host name; a question mark in parentheses (haplotypes DD1 to DD4) indicates uncertainty in number of refractile bodies due to sequences originating from clonal amplicons detected in a single individual host. GenBank accession numbers of the sequences forming the collapsed clades are given in Additional file 1: Table S1. The scale-bar indicates the number of substitutions per site

Bayesian inference and maximum likelihood phylogenetic analyses produced trees with an almost identical topology. All new haplotypes grouped together with *Lankesterella* parasites (Fig. 2). Ten of the 12 new haplotypes, including parasite sequences detected in *U. stansburiana* and *Sceloporus occidentalis*, were included with high phylogenetic support (≥ 95%) in the same clade with sequences of *Lankesterella* parasites (i.e. *Lankesterella* sp. Ae-Lk and *Lankesterella* sp. Lank_anocar) detected in the North American host species, *Anolis carolinensis*, and in the Mediterranean host species, *Acanthodactylus erythrurus* (clade A). The group (named clade B) formed by *L. valsainensis* (detected in Eurasian avian hosts) and the type-species of the genus *Lankesterella*, *L. minima* (detected in Eurasian amphibian hosts), was a sister group to clade A (support < 80%, Fig. 2). In addition, clade C that grouped cloned haplotypes DD1 and DD4 detected in the desert iguana, *D. dorsalis*, from California, formed a third monophyletic clade within the family Lankesterellidae. This last clade was the sister group to the clade formed by the groups A and B with a statistical support of 0.78 by Bayesian inference and 95% by maximum likelihood (Fig. 2). Additionally, clade D contained all previously known sequences of *Schellackia* parasites that were molecularly characterised from blood samples of Asian and European lizard hosts in previous studies [3, 14].

## Discussion

Three *Schellackia* species are known to infect American lizard hosts: *S. landaue*, *S. occidentalis*, and *S. golvani* [4]. We detected parasites that are biologically and morphologically compatible with *S. occidentalis* and *S. golvani*. On the one hand, the sporozoites observed infecting the blood cells of *Anolis carolinensis* are morphologically (i.e. mean length and width [4]) compatible with the original description of *S. golvani*. Besides, the sporozoites were found infecting exclusively polymorphonuclear leukocytes, which is a unique characteristic of *S. golvani* [4, 10]. On the other hand, parasites found in the blood samples of the lizard hosts *Sceloporus occidentalis* and *Uta stansburiana* are likely the same parasite formerly described as *Schellackia occidentalis*. The size (i.e. mean length and width) of the sporozoites found infecting the blood cells of *Sceloporus occidentalis* was compatible with that originally described for the sporozoites of *Schellackia occidentalis* (Table 4) although the number of RB was not always consistent with the original description. However, previous experimental studies demonstrated that RB are dynamic structures within the Eimeriorina that can shift in size and number during the development of the sporozoite and fluctuate in number even in the sporozoites infecting an individual host [4, 40, 41].

**Table 4:** Morphometric data (in micrometres) for sporozoites of known hemococcidian species that infect American lizards and the new haplotypes found in the present study

Parasite haplotype or described species	Host species	*n*	Length		Width	
			Mean ± SD	Range	Mean ± SD	Range
SO1	*S. occidentalis*	96	8.6 ± 1.7	5.1–13.5	4.0 ± 1.1	2.1–10.3
SO2	*S. occidentalis*	14	7.2 ± 0.9	5.7–8.6	3.1 ± 0.6	2.1–3.8
US1	*U. stansburiana*	2	10.1	10.0–10.2	3.3 ± 0.6	2.9–3.7
US2a, US2b	*U. stansburiana*	12	9.3 ± 0.6	8.5–11.0	3.4 ± 0.8	2.1–5.3
US3	*U. stansburiana*	12	9.5 ± 1.9	5.4–12.0	2.6 ± 0.5	1.6–3.7
*Schellackia occidentalis* ^a^	*S. occidentalis* and *U. stansburiana*	–	7.8	5.6–9.6	3.6	2.8–5.6
*Lankesterella* sp. Lank_anocar	*A. carolinensis*	21	8.4	7.1–9.9	3.9	2.6–4.5
*Schellackia golvani* ^a^	*Anolis* spp.	–	8.2	7.6–10.7	3.3	3.1–3.8
DD1, DD2, DD3, DD4	*D. dorsalis*	63	7.9 ± 1.3	5.2–11.3	3.7 ± 0.8	2.2–6.1
LP1	*L. pictus*	4	6.5 ± 0.6	5.9–7.4	3.5 ± 0.7	2.7–4.1
PP1^b^	*P. payuniae*	7	8.8 ± 1.9	7.1–12.7	4.3 ± 0.7	3.1–5.2
*Schellackia landaue* ^a^	*Polychrus* spp.	–	13.0	–	3.0	–
*Lainsonia iguanae* ^a^	*Iguana iguana*	–	11.0	–	7.0	–
*Lainsonia legeri* ^a^	*Tupinambis nigropunctatus*	–	9.5	–	4.0	–

*Abbreviation*: *SD* standard deviation

^a^Data of *Schellackia* species from [4]

^b^Data from [30]

Considering that we included sequences of *S. golvani* and *S. occidentalis* in the phylogenetic analyses, it was unexpected that none of them were closely related to the family Schellackiidae (clade D) [23]. To our knowledge, this phylogenetic study is the first to compare hemococcidian parasites from American, European and Asian lizard species. We found strong phylogenetic support to conclude that the hemococcidian haplotypes detected in American lizard hosts are closely related to the genus *Lankesterella* and distant to the genus *Schellackia*. Indeed, lankesterellids detected in North and South American lizards were more closely related to *Lankesterella* sp. Ae-Lk1 and *Lankesterella* sp. Lank_anocar, previously detected in Old and New World lizards [3, 23] (clade A) than to *Lankesterella* spp. detected in European bird and amphibian hosts (clade B). The phylogenetic affinity of clade C with the remaining clades within the Lankesterellidae showed medium to high phylogenetic support and, hence, it provides molecular evidence for a monophyletic origin of the genus *Lankesterella*.

Based on the phylogenetic affinities achieved here and the phylogenetic support for the monophyletic origin of the genus *Lankesterella*, our recommendation is to reclassify *Schellackia golvani* as *Lankesterella golvani* (Rogier & Landau, 1975) n. comb*.,* and *S. occidentalis* as *L. occidentalis* (Bonorris & Ball, 1955) n. comb*. Lankesterella occidentalis* n. comb. seems to be composed of a diverse complex of haplotypes from parasites that infect different host species in North America (see [4]). Indeed, we characterised six different 18S RNA haplotypes in only two different host species of the family Phrynosomatidae. However, the specificity at the host genus level of American lankesterellids was high because none of the parasite haplotypes detected in *Uta stansburiana* was found in *Sceloporus occidentalis* (and *vice versa*) despite sampling ranges of the hosts and parasites overlapped (e.g. in California). This result suggests that *L. occidentalis* n. comb*.* may be a complex group of cryptic species similar to previous cases of parasitic species complexes [42]. Indeed, the significantly longer and narrower sporozoites of *L. occidentalis* n. comb*.* that infect *U. stansburiana*, in comparison to the sporozoites that infect *S. occidentalis* hosts, suggest that *Schellackia occidentalis* was formerly described based on a complex of species. Future description of the endogenous development of the parasite in the walls of the intestine of its host may provide new species designations in order to split *L. occidentalis* n. comb*.*

We provide evidence that lankesterellids are a molecularly diverse group of parasites in lizard hosts, at least in the Americas. Indeed, we found 12 different haplotypes of these parasites in five host lizard species. The absence of *Schellackia* haplotypes in this sample of New World lizard hosts suggests that the genus *Schellackia* might be restricted to Old World lizards. Nevertheless, further studies should provide a wider sampling including other localities and host species in North and South America to provide additional evidence on the absence of *Schellackia* spp. in the Americas. This might have important conservation implications because the eventual introduction of a novel pathogen into America (i.e. *Schellackia*) through human activity (e.g. pet trade, see [43]) could have a dramatic negative effect on native lizards.

One of the host lizards included in this study, the desert iguana *Dipsosaurus dorsalis* from Boyd Deep Canyon (CA), was infected by at least four *Lankesterella* haplotypes (i.e. DD1-DD4). Two possible scenarios may explain the presence of these four clonal parasite haplotypes in a single host individual. On the one hand, these haplotypes could be copies of the same *Lankesterella* species. Although all known eukaryotes have several clonal copies of the 18S rRNA gene, some apicomplexan species transcribe different copies of this gene along their life-cycle [43]. This is the case for *Plasmodium vivax* (Apicomplexa: Haemosporidia) [44], and it has also been suggested for the coccidian genus *Hepatozoon* [45]. In our sample, the absence of multiple peaks in the chromatograms from the uncloned sequences does not rule out the possibility that multiple copies of the 18S gene were also present. On the other hand, the phylogenetic position of haplotypes D1 and D4 as the sister clade to groups A and B only partially support this assumption. In this sense, the presence of different haplotypes of the 18S rRNA gene in one individual might reflect the presence of a mixed infection with different parasite species. Indeed, many cryptic species may occur within Apicomplexa [42, 46] because hematic stages of unicellular parasites may lack enough diagnostic morphological traits to distinguish easily among species when mixed infections occur [47].

Previous studies on vector-parasite evolution suggest that vector diversity and vector-protozoan specificity may be high in avian models [48]. If that also occurs in lizards, the high genetic diversity of hemococcidians detected in the present study might be related to the high diversity of their vectors, indicating high vector-parasite specificity. A recent publication described high genetic diversity of *Lutzomyia* (Diptera: Psychodidae) sand flies in California [49]. Measures of genetic differentiation indicated genetic differentiation of this vector between sites that were approximately 0.5–3.8 km distant. *Lutzomyia* sand flies, together with some mite species, have been described as natural vectors for several diseases of lizards caused by protozoans including hemococcidia [4, 50, 51]. Thus, the presence of a high genetic diversity of transmitters would favour diversification of host-parasite associations and, hence, diversification of hemococcidian parasites in America.

## Conclusions

Life history traits of hemococcidian parasites such as the type of host blood cells infected, host species or number of refractile bodies are not valid diagnostic characteristics to differentiate the parasites between the genera *Schellackia* and *Lankesterella*. Indeed, *Lankesterella* spp. with a different number of refractile bodies had a close phylogenetic origin. Based on the phylogenetic results we provide a systematic revision of the North American hemococcidians. Our recommendation is to include the species formerly described in the genus *Schellackia* (i.e. *S. golvani* and *S. occidentalis*) that infect American lizards into *Lankesterella* (Lankesterellidae).

## Additional file

Additional file 1: Table S1. GenBank accession numbers for all sequences included in the phylogenetic analyses. Numbers in column C identify collapsed clades in Fig. 2 (DOCX 24 kb)
