# Supplementary material for: Phylogenetic analyses reveal that Schellackia parasites (Apicomplexa) detected in American lizards are closely related to the genus Lankesterella: is the range of Schellackia restricted to the Old World?
Source: Parasit Vectors. 2017 Oct 10;10:470. doi: 10.1186/s13071-017-2405-0 (PMC5633878; doi:10.1186/s13071-017-2405-0)
Supplement: Additional file 1: Table S1. — GenBank accession numbers for all sequences included in the phylogenetic analyses. Numbers in column C identify collapsed clades in Fig. 2 (DOCX 24 kb) [file 13071_2017_2405_MOESM1_ESM.docx]

| **Taxon** | **GenBank** | **C** |  | **Taxon** | **GenBank** | **C** |  |
| --- | --- | --- | --- | --- | --- | --- | --- |
| *Goussia neglecta* | FJ009242 | 7 |  | *Schellackia bolivari* | KJ131416 | 5 |  |
| *Goussia sp.* | FJ009243 | 7 |  | *Caryospora bigenetica* | AF060975 | 3 |  |
| *Goussia noelleri* | FJ009241 | 7 |  | *Caryospora bigenetica* | AF060976 | 3 |  |
| *Eimeria onychomysis* | AF307879 | 1 |  | *Lankesterella minima* | AF080611 | - |  |
| *Eimeria reedi* | AF311642 | 1 |  | *Lankesterella valsainensis* | DQ390207 | - |  |
| *Eimeria leucopi* | AF339491 | 1 |  | *Lankesterella* sp. Lank_anocar | KU180248 | - |  |
| *Eimeria albigulae* | AF307880 | 1 |  | *Lankesterella* sp. Ae-Lk | KJ131417 | - |  |
| *Eimeria chaetodipi* | AF339489 | 1 |  | *Isospora takydromi* | KU180238 | 2 |  |
| *Eimeria peromysci* | AF339492 | 1 |  | *Isospora fahdi* | KU180239 | 2 |  |
| *Eimeria rioarribaensis* | AF307877 | 1 |  | *Isospora abdalahi* | KU180240 | 2 |  |
| *Eimeria chobotari* | AF324214 | 1 |  | *Isospora amphiboluri* | KU180241 | 2 |  |
| *Eimeria dipodomysis* | AF339490 | 1 |  | *Isospora wiegmanniana* | KU180242 | 2 |  |
| *Eimeria antrozoi* | AF307876 | 1 |  | *Isospora albogularis* | KU180243 | 2 |  |
| *Isospora gryphoni* | AF080613 | 1 |  | *Isospora chafarinensis* | KU180244 | 2 |  |
| *Isospora robini* | AF080612 | 1 |  | *Isospora tarentolae* | KU180245 | 2 |  |
| *Atoxoplasma sp.* | AY331571 | 1 |  | *Isospora gekkonis* | KU180246 | 2 |  |
| *Eimeria catronensis* | AF324213 | 1 |  | *Caryospora ernsti* | KU180247 | - |  |
| *Eimeria pilarensis* | AF324215 | 1 |  | *Eimeria tropidura* | AF324217 | 6 |  |
| *Eimeria papillata* | AF311641 | 1 |  | *Choleoeimeria* sp. | AY043207 | 6 |  |
| *Eimeria nieschulzi* | U40263 | 1 |  | *Choleoeimeria gallotiae* | KR360728 | 6 |  |
| *Eimeria falciformis* | AF080614 | 1 |  | *Eimeria eutropidis* | KR360729 | 6 |  |
| *Eimeria sevilletensis* | AF311644 | 1 |  | *Choleoeimeria scincorum* | KR360730 | 6 |  |
| *Eimeria separata* | AF311643 | 1 |  | *Acroeimeria cf. tarentolae* | KR360731 | 6 |  |
| *Eimeria telekii* | AF246717 | 1 |  | *Eimeria steinhausi* | KR360732 | 6 |  |
| *Eimeria langebarteli* | AF311640 | 1 |  | *Choleoeimeria wiegmanniana* | KR360733 | 6 |  |
| *Eimeria scholtysecki* | AF324216 | 1 |  | *Eimeria tokayae* | KR360734 | 6 |  |
| *Eimeria alabamensis* | AF291427 | 1 |  | *Acroeimeria sceloporis* | KR360735 | 6 |  |
| *Eimeria bovis* | U77084 | 1 |  | *Toxoplasma gondii* | L37415 | - |  |
| *Eimeria weybridgensis* | AY028972 | 1 |  | *Neospora caninum* | AJ271354 | - |  |
| *Eimeria meleagrimitis* | AF041437 | 1 |  | *Frenkelia glareoli* | AF009245 | 9 |  |
| *Cyclospora cayetanensis* | AF111183 | 1 |  | *Frenkelia microti* | AF009244 | 9 |  |
| *Cyclospora* sp. | U40261 | 1 |  | *Sarcocystis buffalonis* | AF017121 | 9 |  |
| *Cyclospora papionis* | AF111187 | 1 |  | *Sarcocystis hirsuta* | AF017122 | 9 |  |
| *Cyclospora* sp. | AF061567 | 1 |  | *Sarcocystis cruzi* | AF017120 | 9 |  |
| *Cyclospora* sp. | AF061568 | 1 |  | *Sarcocystis hominis* | AF006470 | 9 |  |
| *Eimeria* sp. | FN298443 | 1 |  | *Besnoitia jellisoni* | AF291426 | - |  |
| *Eimeria adeneodei* | AF324212 | 1 |  | *Besnoitia besnoiti* | AF109678 | - |  |
| *Eimeria tenella* | AF026388 | 1 |  | *Hyaloklossia lieberkuehni* | AF298623 | - |  |
| *Eimeria mitis* | U40262 | 1 |  | *Cystoisospora timoni* | AY279205 | 8 |  |
| *Eimeria cf. mivati* | FJ236378 | 1 |  | *Isospora felis* | L76471 | 8 |  |
| *Eimeria acervulina* | EF210324 | 1 |  | *Isospora suis* | U97523 | 8 |  |
| *Eimeria praecox* | FJ236371 | 1 |  | *Isospora orlovi* | AY365026 | 8 |  |
| *Eimeria brunetti* | U67116 | 1 |  | *Isospora belli* | AF106935 | 8 |  |
| *Eimeria maxima* | EF210322 | 1 |  | *Lankesterella* sp. US3 | MF167544 | - |  |
| *Eimeria reichenowi* | AB205175 | 4 |  | *Lankesterella* sp. DD2 | MF167545 | - |  |
| *Eimeria reichenowi* | AB205170 | 4 |  | *Lankesterella* sp. DD3 | MF167546 | - |  |
| *Eimeria gruis* | AB205165 | 4 |  | *Lankesterella* sp. DD1 | MF167547 | - |  |
| *Eimeria trichosuri* | FJ829323 | - |  | *Lankesterella* sp. DD4 | MF167548 | - |  |
| *Eimeria arnyi* | AY613853 | 5 |  | *Lankesterella* sp. US1 | MF167549 | - |  |
| *Eimeria ranae* | EU717219 | 5 |  | *Lankesterella* sp. SO1 | MF167550 | - |  |
| *Schellackia* sp. Ls-A | JX984674 | 5 |  | *Lankesterella* sp. SO2 | MF167551 | - |  |
| *Schellackia* sp. Ls-B | JX984675 | 5 |  | *Lankesterella* sp. US2a | MF167552 | - |  |
| *Schellackia* sp. Ph-B4 | JX984676 | 5 |  | *Lankesterella* sp. US2b | MF167553 | - |  |
| *Schellackia orientalis* | KJ131414 | 5 |  | *Lankesterella* sp. PP1 | MF167554 | - |  |
| *Schellackia bolivari* | KJ131415 | 5 |  | *Lankesterella* sp. LP1 | MF167555 | - |  |

**Additional file 1: Table S1.** GenBank accession numbers for all sequences included in the phylogenetic analyses. Numbers in column C identifies collapsed clades in Fig. 1
